# Nutrient efficiency at the core of nutrition and sustainability

**DOI:** 10.3389/fnut.2023.1248895

**Published:** 2024-01-05

**Authors:** Serge Rezzi, Christian Nils Schwab, Yiannis Kourmpetis, Martin Kussmann, Stéphane Canarelli, Roger Darioli

**Affiliations:** ^1^Swiss Nutrition and Health Foundation, Epalinges, Switzerland; ^2^Integrative Food and Nutrition Center, École polytechnique fédérale de Lausanne, Lausanne, Switzerland; ^3^Siftlink SA, Epalinges, Switzerland; ^4^Kompetenzzentrum für Ernährung (KErn), Freising, Germany; ^5^Kussmann Biotech GmbH, Nordkirchen, Germany

**Keywords:** nutrition, nutrient, nutrient bioavailability, nutrition efficiency, health, sustainability, life cycle assessment, circularity

## 1 Introduction

The nutrition health benefits are determined by the fractions of macronutrients, micronutrients, and phytonutrients, either in their intact or metabolized forms, that reach the sites of action in the body where they are expected to fulfill the needs. These benefits are thus proportional to the efficiency with which dietary nutrients are turned into their respective biologically available and active molecules. Over the last decades, the understanding of the health benefits of dietary proteins has evolved from the consideration of daily recommended intake (essentially as bulk protein) via their amino acid profile and quality (including protein digestibility and biological value) to—more recently—their potential to release bioactive peptides during digestion (1, 2). The term “efficiency” has been related to dietary proteins through the introduction of the “protein efficiency ratio,” a measurement of the growth-promoting value of a protein, and through “efficiency of protein utilization” (1, 3, 4). However, we suggest that the concept of “protein efficiency” should extend across the full functionality spectrum of proteins from meeting amino acid requirements via modulating the immune system and microbiome, to improving micronutrient absorption and generating bioactive peptides. By extension to all macro-, micronutrients and phytonutrients, the concept of “nutrient efficiency” can thereby express the fraction of dietary nutrients that can effectively contribute to meeting nutritional requirements and nutrition-associated health benefits. In doing so, nutrient efficiency can contribute to bridging nutrition and sustainability concepts.

In the 20^th^ century, a major energy sustainability achievement was the introduction of light-emitting diodes (LEDs) into a broad range of daily used lighting systems and electronic devices. Enabled by advances in quantum physics and semiconductors, lighting technology evolved from incandescent lamps to modern LEDs that show longer lifespan and consumption of 75% less energy for an equivalent light emission. This important achievement is primarily due to the technological improvement of the efficacy of energy conversion from electrons into the wanted functionality via photon production. By analogy, nutrition sustainability concepts and related definitions would benefit from a clarification about the efficiency with which dietary nutrients are converted into their respective bioavailable compounds to adequately nourish the human body. This “cellular nutrition” concept encompasses eukaryotic cells and symbiotic microorganisms involved in promoting healthy growth and development as well as preventing malnutrition, e.g., undernutrition, and reducing diet-associated risks of non-communicable diseases.

Different concepts and definitions have been proposed in a legitimate attempt to integrate nutrition, health and sustainability related to food systems and environment. In 2010, the Food and Agriculture Organization (FAO) of the United Nations defined sustainable diets as “those diets with low environmental impacts which contribute to food and nutrition security and to healthy life for present and future generations. Sustainable diets protect biodiversity and ecosystems, are culturally acceptable, accessible, economically fair and affordable; nutritionally adequate, safe, and healthy, while optimizing natural and human resources” (5). In 2011, the concept of “nutrition ecology” was introduced with the goal of integrating the food supply chain with the multiple dimensions of health, environment, society, and economy (6). A few years later, “nutritional sustainability” was defined as “the ability of a food system to provide sufficient energy and the amounts of essential nutrients required to maintain good health of the population without compromising the ability of future generations to meet their nutritional needs” (7). Although this concept of “nutritional sustainability” was originally introduced for pet foods, authors duly report on the importance of nutrient quality, digestibility, and bioavailability. The terminology continued to evolve with the introduction of “sustainable nutrition security” which is based on seven metrics including food nutrient adequacy, ecosystem stability, food affordability and availability, socio-cultural wellbeing, food safety, resilience, as well as waste and loss reduction (8). More recently, the terminology of “nutritional sustainability” was revisited by Smetana et al. (9) with the aim to estimate the environment's capacity within defined planetary boundaries through modifiable components for the food system transformation. In 2019, the FAO and the World Health Organization (WHO) have joined efforts to provide convergence of the different concepts of sustainable and healthy diets with 16 guiding principles for Sustainable Healthy Diets (SHD) (10). Amongst those principles, SHD are “adequate (i.e., meeting but not exceeding needs) in energy and nutrients for growth and development, and to meet the needs for an active and healthy life across the lifecycle” and “consistent with WHO guidelines to reduce the risk of diet-related NCDs, and ensure health and wellbeing for the general population” (11). The notion of “dietary adequacy” is derived from nutrient recommendations that are defined from food nutrient compositions and population-based nutrient requirements. Although pragmatic, such a reductionist approach to defining nutrient and health adequacy struggles with capturing the inherent multidimensionality of the interactome between nutrients and health outcomes. First, the body's exposure to nutrients is determined by diverse historical, religious, social, cultural, and economic factors as well as the consciousness about environmental and animal welfare. This by itself can result in an infinite number of possible dietary patterns at the individual level. On the other hand, the actual ability to digest and metabolize nutrients is determined by both the inter- and intra-individual biological variability, but also by the composition of dietary patterns and the type of food processing.

We believe that the provided definitions and concepts about nutrition and sustainability can benefit from a simplified and integrative terminology able to qualify, beyond food nutrient composition and/or density, the ability of foods to provide the human body with nutritionally usable nutrients to deliver SHD. We hence propose a new concept of “nutrient efficiency” that can properly account for the principles of nutrient bioaccessibility, digestibility, bioavailability, and adequacy to requirements that remain poorly understood and communicated. Nutrient efficiency expresses, for each nutrient in each food product, the fraction of nutrient intake that effectively contributes to meeting the nutritional requirements as defined by age, gender, physiological status, or specific health/disease conditions.

## 2 Nutrient efficiency of plant-based foods

The usefulness of the nutrient efficiency concept can be exemplified by the nutritional qualities of plant-based foods. Whereas the need for alternative protein sources is primordial for both planetary and human health, plant-based foods often show nutritional gaps, particularly with regard to protein quality. Yet, many plant-based foods are promoted and commercialized as “sustainable and healthy alternative protein” thereby possibly misguiding the consumer in terms of protein and amino acid sufficiency. Moreover, packaging labeling often attributes rather favorable nutritional scores to plant-based foods, which may not sufficiently take into account the issue of protein quality. The nutritional quality of a protein is defined as its capacity to meet metabolic needs in terms of amino acids and nitrogen, particularly considering protein amino acid composition, digestibility, and human nutritional requirements (1, 12, 13). Besides those protein characteristics, it has been proposed to extend protein quality to their broad range of biological functions in the body (13). Different metrics have been developed for the determination of protein quality with the digestible indispensable amino acid score (DIAAS) that is nowadays recognized as the standard by the FAO (1).

Furthermore, plant-based foods may also contain anti-nutritional factors that can decrease protein digestion (protease inhibitors) and, therefore, protein quality, alter the integrity of the intestinal barrier (lectins), and limit absorption of several micronutrients such as iron and calcium (phytates, tannins, oxalates). Despite available scientific evidence, such nutritional limitations of plant-based foods remain incompletely communicated to consumers in favor of a sustainability communication limited to food ecological aspects. Notably, this directly relates to nutrient efficiency as a partial digestibility of plant proteins implies a sub-optimal conversion of dietary proteins into amino acids, particularly essential amino acids, to meet the needs for endogenous protein synthesis and metabolic demand in humans. On the other hand, the undigested protein fraction is further metabolized by the gut microbiome via putrefaction reactions. Whereas microbial putrefaction plays a key role in maintaining a mutually beneficial, i.e., symbiotic host-microbiome relationships, we need to scientifically establish whether excessive microbiome exposure to partly digested proteins may with time result in yet unknown dysbiosis and disease onset (14, 15).

## 3 Limitations and opportunities for plant-based foods

Technological solutions and operational capabilities of the food production system must meet the global demand for nutritionally adequate products. Yet, industrial production of plant-based foods with optimized sustainability and nutritional value must find a balance between consumer expectations (price point, food hedonics, clean label), security of key ingredients, food processing and upscaling requirements. Plant-based foods are often ultra-processed food (UPF) products that trigger debates about possibly poor diet quality and related health concerns (16–18). Recent results from systematic and meta-analyses investigating relationships of high and low UPF consumption with disease and mortality outcomes reveal public health concerns. Effect size of high UPF consumption shows significant statistical associations with increased incidence of arterial hypertension (19), diabetes (20, 21), and cardio- or cerebrovascular diseases (22, 23), overweight and obesity (23, 24), as well as mental disorders, anxiety and depression (23, 25, 26). Furthermore, associations of high UPF consumption with either cardio- or cerebrovascular (23) or all-cause mortality (22–24, 27) were also reported. However, these associations originate from observational studies with their intrinsic limitations and require thus proper scientific validation to establish causality and molecular mechanisms linking consumption of UPF products and health outcomes. However, it is assumed that high UPF consumption associates with unbalanced nutrient intakes, with increased exposure to sugars and high glycemic load products, sodium, saturated and trans-fats, as well as food additives. High UPF consumption can also associate with reduced intake of micronutrients, fibers, and healthy foods.

Current industrial design of plant-based foods that begins with the choice of key ingredients such as protein isolates may favor intensive food processing to meet palatability requirements. This ingredient-based approach is reductionist and under-valorizes the nutritional potential of the whole plant resource while generating significant volumes of co- and byproducts. It essentially relies on the cracking of foods into their constitutional components to re-aggregate them into processed foods creating sustainability impacts along the value chain. Within planetary environmental and food resource boundaries, industry adoption of more sustainable processes is necessary to deliver safer, more palatable, less processed, yet nutrient-efficient foods. The bioguided food process, exemplified with human milk, is based on a food process design that benefits from a detailed knowledge of the raw material composition and structure with the aim to optimize the nutritional characteristics of processed food (28). Enhanced nutritional properties through improved nutrient bioaccessibility and bioavailability should be considered as input variables in the food process design. They should be properly communicated and regulated by governmental bodies with the support of scientific authorities. Furthermore, such innovative food processes can deploy and leverage enzymatic and fermentation capabilities of microorganisms, including bacteria, yeast, and fungi to produce specific nutrients (precision fermentation) or to modify the structure, composition, and taste properties of foods. For example, lacto-fermentation is a natural and effective process to improve the nutritional properties of plant-based foods that takes advantage of both catabolic and anabolic capabilities of microorganisms to reduce anti-nutrients and improve digestibility and bioaccessibility of nutrients and micronutrients (29).

Combinatorial possibilities based on nutritionally adequate plant raw materials (used alone or in combinations) and minimally invasive processing techniques (soaking, germination, heating, fermentation) open an entire field of opportunities to develop food products with improved nutrient efficiency and environmental impact minimizing nutrient loss and waste. Exploration of this space of possibilities might be facilitated by the rapidly developing domain of artificial intelligence (AI). Recent advances in AI and Deep Learning have enabled several breakthroughs in biology and drug discovery (30, 31) and will eventually affect nutrition science and food technology as well. AI may prove more powerful than classical data science and sole statistics in modeling the complex interdependencies between nutrient efficiency, food safety, hedonics, pricing, and environmental impact (especially minimizing energy use and by-product generation). Such AI models might be used to propose new food processing strategies, including combinations of complementary raw materials in terms of nutrient profiles and mixed-strain fermentation or thermal treatment parameters that can lead to new natural, clean-label, and nutritionally improved plant-based products (32).

## 4 “Nutrient efficiency” to support sustainable healthy diets

Animal proteins (beef, chicken, fish, egg, milk) often show higher nutritional values for humans than plant-based proteins but their production is most impactful on environment. To meet the increased protein demand driven by global population growth, alternative and sustainable sources and productions need to be developed. Together with reduction of meat consumption, new production options, using for instance plant materials, insects, aquaculture, or macro- and microalgae would have to be prioritized as per their ability to attenuate the trade-off between planetary and human health (33). Scalable strategies to improve protein quality via modulation of digestibility and compensation for limiting amino acids invite us to explore minimally invasive, natural, and bioguided food processing approaches on plants alone or in combinations. The “nutrient efficiency” concept extends the quality dimension of proteins to the optimal trade-off between nutritional relevance and environmental impacts of protein production and usage in foods. It represents a base for new integrative approaches designed to measure the yield of nutritional value relatively to environmental sustainability metrics such as life cycle and circularity assessments with the aim to comprehensively account for the impacts of the food systems on biodiversity, water and energy reserves, as well as on greenhouse gas emissions throughout the food value chain until consumer usage (34). The “nutrient efficiency” concept should leverage any opportunity for a circular management of the nutrient reserves including the upcycling of agricultural and industrial co-products into valuable nutritional applications. It could help operate a paradigm shift of food industry toward food processing streams guided by nutrient-preservation, minimal processing without negative food safety consequences, reduction of antinutrients and wastes at minimal energy cost and environmental footprint. The prioritization of bioguided process streams (e.g., fermentation) over the classical use of (rather negatively perceived) ultra-processing methods requiring non-natural additives may facilitate the delivery of natural, clean-label, palatable, sustainable, nutritious and bioactive products, therefore a food production guided by nutrient efficiency.

Beyond proteins, the “nutrient efficiency” concept is applicable to macronutrients, micronutrients, phytonutrients or other health-relevant food bioactives. The concept of “nutrient efficiency” can help assess food items and diets based on the biologically usable nutrient fraction (i.e., fraction effective to meet nutritional requirements) vs. available sustainability metrics or scoring systems. We believe a key strength of the “nutrient efficiency” terminology lies with its ability to capture the health relevant outcome of nutrients vs. sustainability components with no simplistic reduction of the complex, and yet not fully understood, science that governs nutrient bio-accessibility, -availability and -efficacy at cellular and organismal levels. Also, by analogy with the nowadays popular notion of energy efficiency, it is likely that “nutrient efficiency” may benefit from an almost intuitive conceptual understanding by the public, regulatory bodies and policy makers, which is pivotal to succeed in the guidance toward healthier food choices minimizing environmental impacts. “Nutrient efficiency” could therefore be advantageous in supporting SHD initiatives and communication. In an environment of ever-increasing tension between a growing world population and vulnerable supply lines (water shortages, geopolitical tension, reduced productivity due to climate warming), nutrient efficiency' concept, which is directly related to the efficient use of nutrient resources, could also be of paramount importance from a nutrient security perspective.

## Author contributions

SR created the concept of the paper and wrote the manuscript. CS, YK, SC, MK, and RD have contributed to draft and critically revise the manuscript. All authors read and approved the final manuscript.
